# Multi-omics analysis identifies UBA family as potential pan-cancer biomarkers for tumor prognosis and immune microenvironment infiltration

**DOI:** 10.3389/fimmu.2025.1510503

**Published:** 2025-02-17

**Authors:** Haibin Wang, XinLi Liu, Hesen Huang, Meng Tang, Jiwei Li, Tingting Huang, Shengjie Wang

**Affiliations:** ^1^ Department of Gastrointestinal Oncology Surgery, The First Affiliated Hospital of Xiamen University, School of Medicine, Xiamen University, Xiamen, China; ^2^ Department of Medical Oncology, Xiang’an Hospital of Xiamen University, School of Medicine, Xiamen University, Xiamen, China; ^3^ Department of Otolaryngology-Head and Neck Surgery, Xiang’an Hospital of Xiamen University, Fujian, Xiamen, China; ^4^ Department of Laboratory Medicine, West China Hospital of Sichuan University, Chengdu, China; ^5^ Department of Respiratory, Critical Care and Sleep Medicine Xiang’an Hospital of Xiamen University, School of Medicine, Xiamen University, Xiamen, China; ^6^ Department of Medical Oncology, Xiamen Key Laboratory of Endocrine-Related Cancer Precision Medicine, Xiamen, China; ^7^ Department of Thyroid and Breast Surgery, Xiamen Humanity Hospital Fujian Medical University, Xiamen, Fujian, China

**Keywords:** pan-cancer, UBA1, UBA6, prognosis, immune infiltration

## Abstract

**Background:**

UBA1 and UBA6 are classic ubiquitin-activating E1 enzymes, which participate in the ubiquitination degradation of intracellular proteins and are closely related to the occurrence and development of various diseases and tumors. However, at present, comprehensive analysis has not been used to study the role of UBA family in cancers.

**Methods:**

We extracted the relevant data of cancer patients from the TCGA database and studied the relationship between the expression patterns of UBA family and the survival rate, and stage of patients in pan-cancer, especially breast cancer (BRCA), colorectal cancer (COAD), renal cancer (KIRC) and lung adenocarcinoma (LUAD). In addition, we also evaluated their impact on immune infiltration using TISIDB database and R packages.

**Results:**

UBA1 and UBA6 are highly expressed in most cancer types, which may be associated with poor prognosis of patients. This study also investigated their expression had a closely tie with clinical stages in some specific tumors. Furthermore, this study also demonstrated that these genes were closely related to immune score, immune subtypes and tumor infiltrating immune cells.

**Conclusions:**

Our study demonstrated that the differential expression of the UBA family, along with their associated survival landscape and immune infiltration across various cancer types, holds potential as biomarkers linked to cancer immune infiltration. This finding offers a novel perspective for informing the direction of cancer treatment strategies.

## Introduction

Ubiquitin-proteasome system (UPS) mediates more than 80% of protein degradation in eukaryotic organisms (1). Its abnormal function will affect the process of cell proliferation and differentiation (2, 3), DNA repair (4, 5), immune inflammation (6) and signal transduction (7). It is closely related to the occurrence and development of malignant tumors (8, 9), cardiovascular diseases (10), neurodegenerative diseases (11), and is one of the important targets of treatment. Ubiquitin or ubiquitin-like protein covalently binds to the substrate protein under the cascade catalysis of Ubiquitin-activating enzyme E1, Ubiquitin-binding enzyme E2 and Ubiquitin-protein ligase E3, and changes its structure, function, location, metabolism (12, 13), etc., which is one of the important post-translational modifications of protein (14). Treatment for UPS is a new treatment strategy that can improve the prognosis of cancer patients (15).

Classic E1 enzymes include UBA1 and UBA6. UBA1 is the most common ubiquitin activating enzyme, which participates in the ubiquitination of most proteins in the body. David et al. found (16) that the mutation of UBA1 in somatic cells can lead to VEXAS syndrome: vacuoles, E1 ubiquitin-activating enzyme, X-linked, autoinflammatory disease, which is an adult systemic autoimmune disease with hematological manifestations. In addition, previous studies have shown that the abnormal expression of UBA1 is related to the malignant phenotype of lung cancer (LC) (17), liver cancer (18), colorectal cancer (19) and other diseases, and can be used as a potential marker for cancer diagnosis and prognosis. Aaron et al. showed that (20), after UBA1 knockdown, the ubiquitin protein in leukemia and myeloma cells decreased and cell death increased; In animal experiments, inhibiting the expression of UBA1 significantly reduced the weight and volume of tumor. Liu (21) and others found that the ubiquitination of protein in Glioblastoma multiform (GBM) decreased after inhibition of UBA1, which then induced endoplasmic reticulum stress and unfolded protein response, and inhibited the survival, proliferation and colony formation of GBM cell lines and primary GBM cells.

UBA6 only exists in vertebrates and sea urchins, and has 40% sequence homology with UBA1, with double specificity, and can activate ubiquitin and ubiquitin-like protein (Ubl) FAT10 (22) at the same time. UBA6 is the only E1 enzyme that catalyzes FAT10. The tumor suppressor protein p53 is the substrate of FAT10. The double negative regulation of FAT10 and p53 plays a key role in the control of tumorigenesis, and the low level of UBA6 makes the tumor more immunogenic and increases the drug sensitivity of the tumor, which makes UBA6 a potential target for treating diseases (23, 24). The lack of UBA6 in neurons during mouse embryonic development leads to changes in neurons in hippocampus and amygdala, decreased density of dendritic spines, and many behavioral disorders, which are embryolethal (25, 26). In addition, the expression level of UBA6 in T cells increased, but it was lower or not expressed in dendritic cells, macrophages, B cells and natural killer cells (27). Lee et al. showed that UBA6 deficiency in T cells caused intracellular IFN- γ expression increased, and then caused multiple organ inflammation in mice (27).

In conclusion, UBA1 and UBA6 are critically involved in various diseases and cancers. This study investigated the association between the expression levels of UBA1 and UBA6 in diverse tumor types and their implications for diagnosis and prognosis. Our findings indicate that the differential expression of UBA1 and UBA6 is significantly correlated with patient survival rates, tumor grade, and cancer stage. Besides, the immune infiltration analysis of UBA members was also conducted in the study, and these results suggested that UBA1 and UBA6 might serve as independent biomarkers for future clinical precision treatment.

## Materials and methods

### Cancer genome map pan-cancer data

We used UCSC Xena (https://xenabrowser.net/) to download pan-cancer data from the TCGA database, including survival statistics, clinical data, stemness scores (RNA-based), and immunological subtypes (28). Perl software incorporated the UBA1/6 expression. We utilized the Wilcox test to compare normal and malignant tissues. p value less than 0.05 is statistically significant. The “ggpubr” and “pheatmap” R packages displayed the heat map and box diagram, respectively. In addition, the “corrplot” R package was used to analyze the association between the UBA1/6 genes.

### Clinicopathological characteristics and survival analysis of UBA1/6 expression

The UALCAN database (http://ualcan.path.uab.edu/index.html) contains clinical data of various cancer kinds, which can be queried to determine the relative expression of one or more genes and their influence on the survival time of cancer patients in various tumor subgroups and normal tissue (29). In this study, we examined the UBA1/6 mRNA expression level difference between specific cancer and normal tissues using the UALCAN database, *P<0.05, **P<0.01, ***P<0.001.

The Human Protein Atlas (HPA, https://www.proteinatlas.org) database provides a database of immunohistochemical expression in normal and malignant tissues (30). In this investigation, immunohistochemical staining of UBA1/6 protein in normal and BRCA, COAD, KIRC, and LUAD tissues was obtained and evaluated by the HPA database.

In addition, through TISIDB database (http://cis.hku.hk/TISIDB/index.php), the boxplots of UBA1/6 expression at various phases of pathology were obtained (31). The UBA1/6 survival analysis was used for the “survival” and “survival” R packages. P value less than 0.05 is considered as difference. Meanwhile, we collected pan-cancer mRNA expression and survival data from TCGA database for Cox analysis in order to understand the link between UBA1/6 expression and patient survival. Besides, we also detected the prognostic value of UBA1/6 from GEO datasets via Km-Plotter site.

### The SNV and CNV mutational landscape based on GSCA

Gene Set Cancer Analysis (GSCA) database is an integrated platform for analyzing genomic mutational landscape (http://bioinfo.life.hust.edu.cn/web/GSCA/) (32). Based on CNV/SNV module, the proportion of UBA1/6 heterozygous/homozygous and amplification/deletion, Spearman correlation between RBX1/2 mRNA expression and CNV, and the survival difference between their CNV and wild type were displayed in pan-cancer. SNV: single nucleotide variants. CNV: Copy number variations.

### Correlation between the expression of the UBA1/6 gene and pan-cancer immune components

Evaluation the relationship between target genes and tumor microenvironment (TME) usually employs the following indicators: immune score, estimate score, stromal score, DNAss, RNAss and tumor purity. In this study, the Spearman method and “limama” package were used to determine the relationship between UBA1/6 expression and DNA stemness score (DNAss) and RNA stemness score (RNAss). Using the “Estimate” and “limma” R packages with TCGA expression data, the matrix score, immune score, and estimated score of various tumor patients were analyzed. Additionally, TISIDB database was also used for the detection of the association between UBA1/6 expression and immune subtype, and the spearman test was applied for this statistical analysis. CIBERSORT algorithm was used to assess the relationship between UBA1/6 expression and relative proportion of 22 types of immune cells invading each tumor sample. Besides, the link between the UBA1/6 gene and representative immune checkpoints was determined with the help of the spearman correlation test. The correlation between UBA1/6 expression and TMB/MSI was computed by R program, and the Fmsb R package was applied for visualization. *p <0.05, ** p <0.01, *** p <0.001.

### Single-cell analysis of UBA family

Firstly, the corresponding single-cell data in.h5 format and annotation results were downloaded from TISCH platform. Then, using the R software MAESTRO and Seurat to process and analyze the single-cell data. Finally, t-SNE method was employed for re-clustering the immune cells.

### Protein-protein interaction network and GO functional enrichment analysis

GeneMANIA (http://genemania.org) is a user-friendly online resource that enables researchers to investigate the roles and interactions of important genes or genomes. GeneMANIA has 660554667 interactions and 166691 genes from 9 different species (33). In this study, we investigated the Homo sapiens proteins that interact with UBA1/6 and created a PPI network using GeneMANIA. Furthermore, “clusterProfiler” package was employed for investigating the biological process of the UBA1/6 related proteins.

### The correlation analysis between UBA1 and UBA6

Gene expression profiling interactive analysis (GEPIA) is a web tool for the evaluation of the relationship of UBA1/6 in this study.

### Drug sensitivity correlation analysis of UBA1/6 gene

The CellMiner database (https://discover.nci.nih.gov/cellminer/home.do) was used for drug sensitivity analysis of UBA1/6, and the “input,” “lima,” “ ggpubr,” and “ggplot2” R packages were employed for data processing and visualization (34). p less than 0.05 is considered as statistically significant.

### Cell culture and qRT-PCR analysis

Normal colonic epithelial cells and renal tubular epithelial cell (HCoEpiC and HK-2), three colon cancer cells (SW480, SW620 and HCT116) and three renal cancer cells (Caki-1, 786-O and 769-P) were obtained from the American Type Cultural Collection (ATCC) and cultured based on the manufacture instructions. The medium used in the culture process was purchased from the Procell Life Science&Technology Co., Ltd (China).

Until the cell density reaches 80%, the total RNA was extracted and followed analyzed UBA1/6 gene expression. cDNA reverse transcription was explored using the SPARKeasy cellular RNA extraction kit (AC0205, Shandong Sparkjade Biotechnology Co., Ltd.) and the Evo M-MLV RT Kit (AG11711, Accurate Biotechnology (Hunan) Co., Ltd. (Changsha, China)). Meanwhile, fluorescence quantitative PCR amplification was performed using 2x SYBR Green qPCR Mix (AH0103-A), purchased from Shandong Sparkjade Biotechnology Co., Ltd. The 2^-ΔΔCT^ formula was employed for measuring the relative expression value of target genes. One-way ANOVA test was applied for evaluating the UBA1/6 relative expression and *p < 0.05, **p < 0.01, ***p < 0.001. ns: no statistically significant. The primers used were as follows: UBA1 forward, 5′-GGTCAAGGCTGTTACCCTACA-3′,

UBA1 reverse, 5′-CGGTTTTTACCGATGTCCTCC-3′;

UBA6 forward, 5′-GGGACTGGCAGCACAAATAAA-3′,

UBA6 reverse, 5′-TCTCCAAGAACGTACCTCTGTC-3′.

## Results

### Expression of UBA1/6 in various types of cancers and their association with pathological features

**Figure 1:**
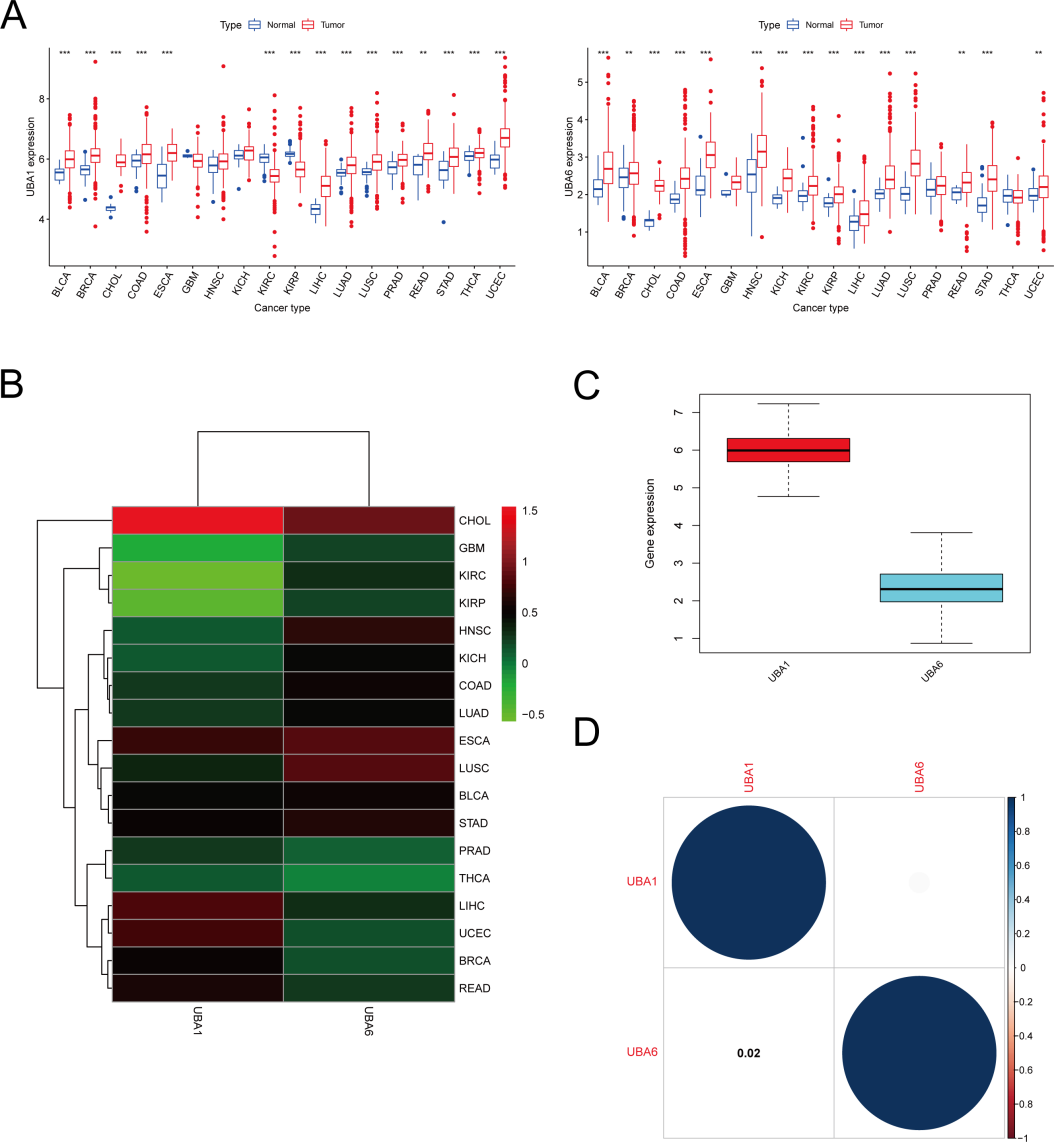
mRNA expression pattern of UBA1/6 in cancers. **(A)** Comparison of UBA1/6 expression between tumor and normal samples. **(B)** Heatmap showing the difference of UBA1/6 gene expression among 18 cancer types in the TCGA database. Red and green represent high or low expressions, respectively. **(C)** The box diagram shows the distribution of UBA1/6 gene expression in various cancers. **(D)** Correlation between UBA1 and UBA6. The blue dot indicates a positive correlation. **p<0.01, ***p<0.001.

We performed a scale analysis of UBA1/6 expression in the TCGA database and found that they were highly expressed in most cancers. However, there were some significant peculiarities in 18 cancers, where UBA6 expression was significantly increased in both HNSC and KICH (P<0.001) and not different in UBA1 (P>0.05) when compared with normal tissues, while the opposite was true in PRAD and THCA, where UBA1 expression was significantly increased in both (P<0.001), while in UBA6 there was no difference (P>0.05). In contrast to normal tissues, a significant decrease in UBA1 expression (P<0.001) and a significant increase in UBA6 expression (P<0.001) were observed in both KIRC and KIRP (**Figure 1A**). Further analysis showed differences in UBA1/6 gene expression in tumors compared to adjacent normal tissues. For example, UBA6 expression was higher in both HNSC and LUSC tissues than in adjacent non-HNSC and non-LUSC tissues, whereas the opposite was true for UBA1. UBA1 expression was higher in both LIHC and UCEC tissues than in adjacent non-LIHC and non-UCEC tissues, whereas the opposite was true for UBA6. (**Figure 1B**). In pan-cancer, the total expression level of UBA1 was higher than that of UBA6 (**Figure 1C**). We also analyzed that there was no significant correlation between the two genes UBA1 and UBA6 (correlation coefficient = 0.02, **Figure 1D**).

**Figure 2:**
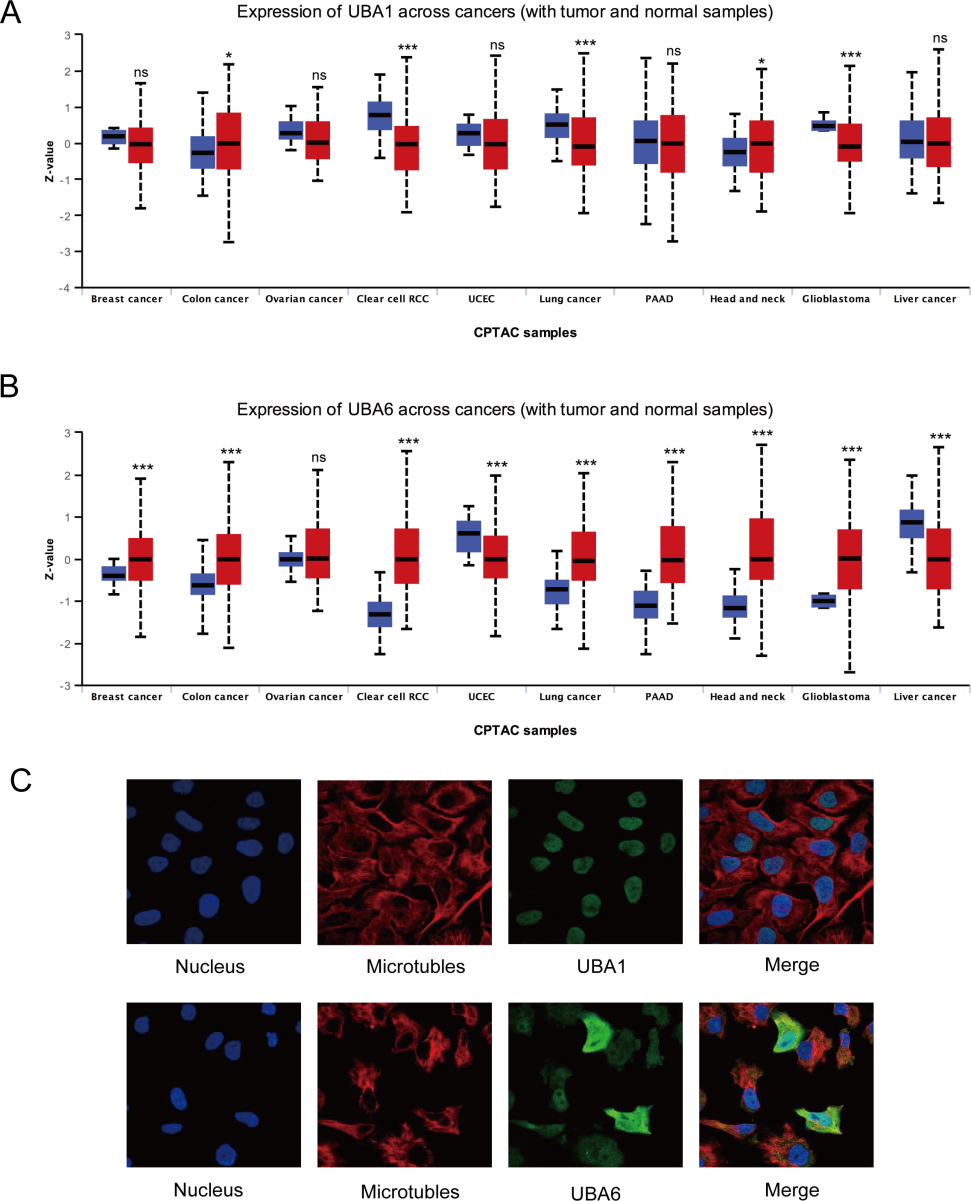
The protein landscape of UBA1/6 in the several specific tumor types. *p < 0.05, ***p < 0.001, ns means no statistical difference **(A, B)**. **(C)** Subcellular localization of UBA1/6 protein. Immunofluorescence images showing intracellular localization of UBA1 in U-2OS and UBA6 in Hela cells.

Aiming for investigation the protein patterns of UBA1/6 in various tumors, the UALCAN database was used in this study to detect their protein level, and the results suggested that UBA6 was highly expressed in most cancers, while the protein expression of UBA1 was lower in renal cell cancer, lung cancer and glioblastoma than corresponding normal samples (**Figures 2A, B**). In contrast to gene expression, the protein level of UBA1 was more strongly expressed in lung cancer, suggesting UBA1 may preferentially enhance its ubiquitination properties within the lung cancer microenvironment, thereby modulating its own stability. Furthermore, immunofluorescence results showed that UBA1 was mainly localized in the nucleoplasm in U-2OS, which provides a physical basis for it to perform ubiquitination, however, UBA6 was detected in the cytosol as well as in the nucleoplasm (**Figure 2C**).

**Figure 3:**
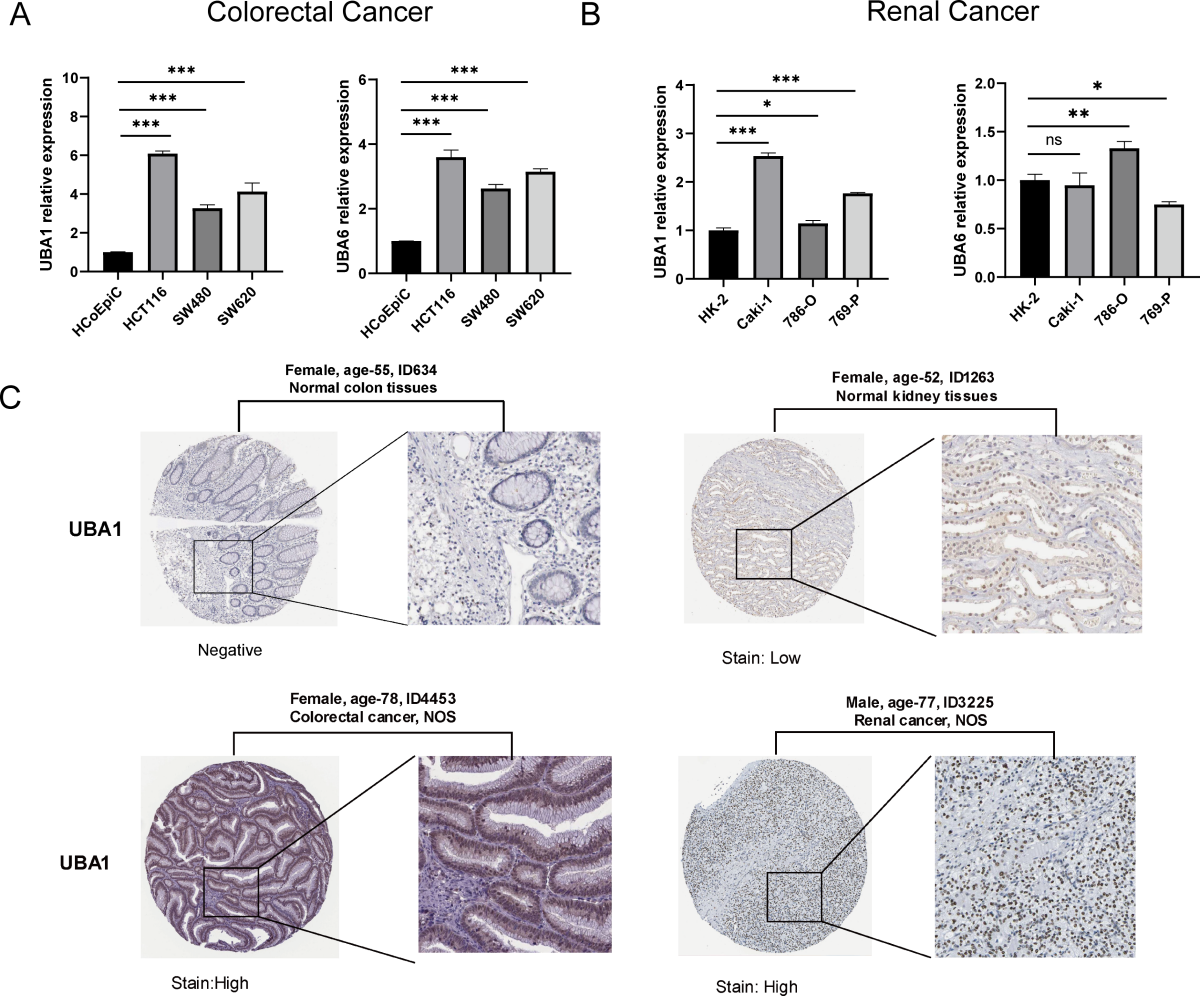
Relative mRNA levels of UBA1/6 in specific two types of cancer. **(A)** Colorectal Cancer, and **(B)** Renal Cancer. The experiments were repeated three times. ^∗^p < 0.05, ^∗∗^p < 0.01, and ^∗∗∗^p < 0.001. ns, no statistically significant. **(C)** The protein level of UBA1 expression in colorectal cancer and renal cancer tissues and matched normal tissues from the HPA database.

**Figure 4:**
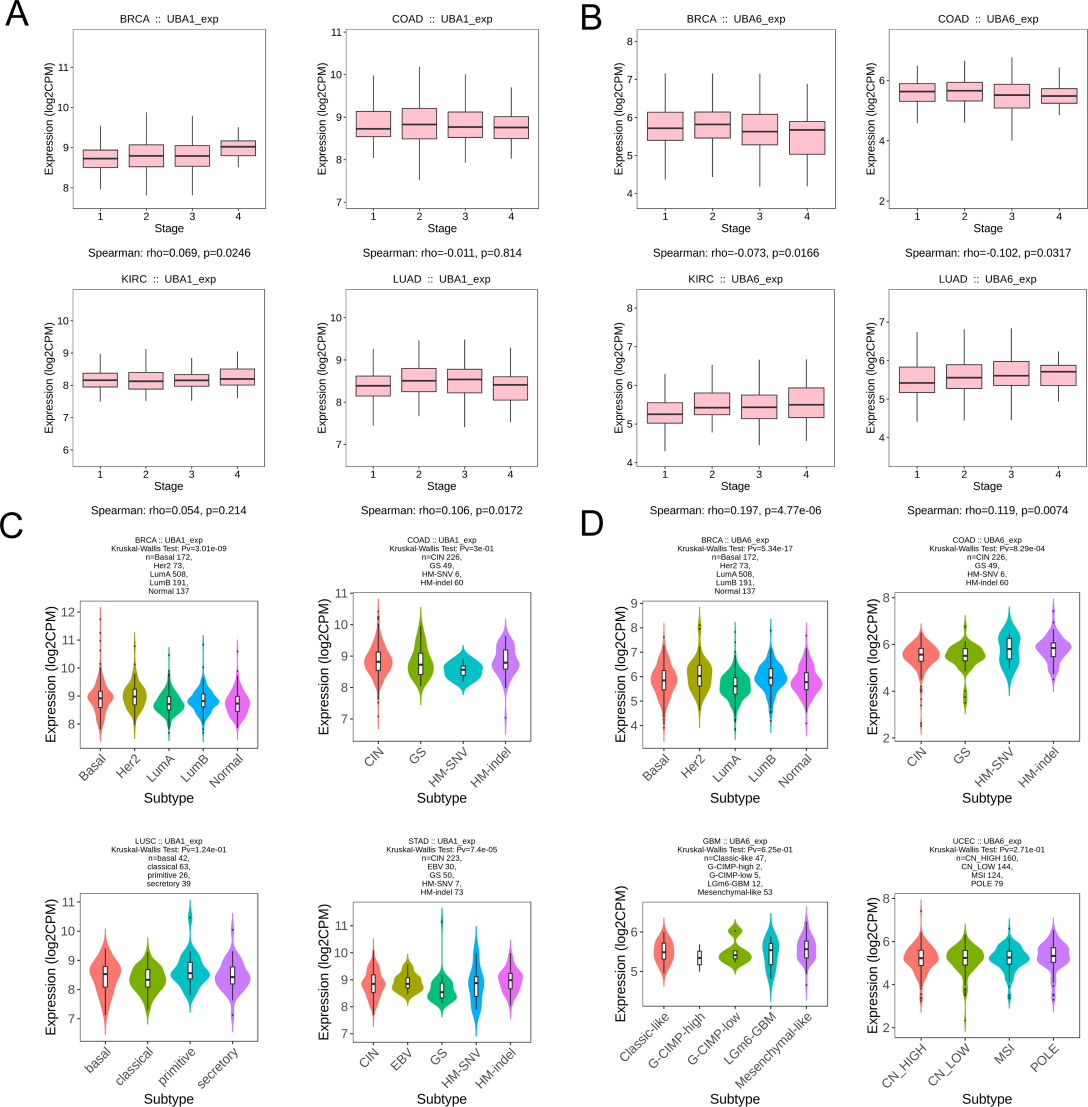
Association with UBA1/6 expression and individual clinical pathological features. **(A, B)** The relation between UBA1/6 expression and pathological stages (stage I, stage II, stage III, and stage IV) of BRCA, COAD, KIRC, and LUAD. **(C, D)** The relation between UBA1/6 expression and pathological subtypes of BRCA, COAD, KIRC, and LUAD. p value less than 0.05 is considered a difference.

To verify the difference in UBA1/6 expression, we analyzed the transcriptional expression of both genes in various tumor cell lines from two common cancers (COAD and KIRC) and normal cells using qRT-PCR experiment, and the result suggested that UBA1 was highly expressed in COAD and KIRC cell lines compared with matched normal cells, while UBA6 was only highly expressed in 786-O cell, compared to normal tubular epithelial cells (**Figures 3A, B**). From the HPA database, we indicated that the protein expression level of UBA1 in colorectal cancer and renal cancer tissues was greater than that in matched normal tissues (**Figure 3C**), but there was no significant difference in the protein expression of UBA6 (**Supplementary Figure S1**), which may reveal that UBA1 was more likely to be a promising prognostic factor in these tumors. Additionally, we also utilized the TISIDB database to demonstrate the link between UBA1/6 expression and the pathological stages and clinical subtypes of numerous malignancies, including BRCA, COAD, KIRC, and LUAD (**Figures 4A, B**). UBA1/6 expression was significantly correlated with both BRCA and LUAD stages. UBA6 was significantly correlated with COAD and KIRC, while UBA1 was the opposite. Besides, UBA1 expression was related with BRCA and STAD, and UBA6 in BRCA and COAD (**Figures 4C, D**). These results revealed to some extent that different expression patterns of UBA1/6 in different cancer types may lead to different tumor phenotypes.

### Prognostic value of UBA1/6 for different cancer types

**Figure 5:**
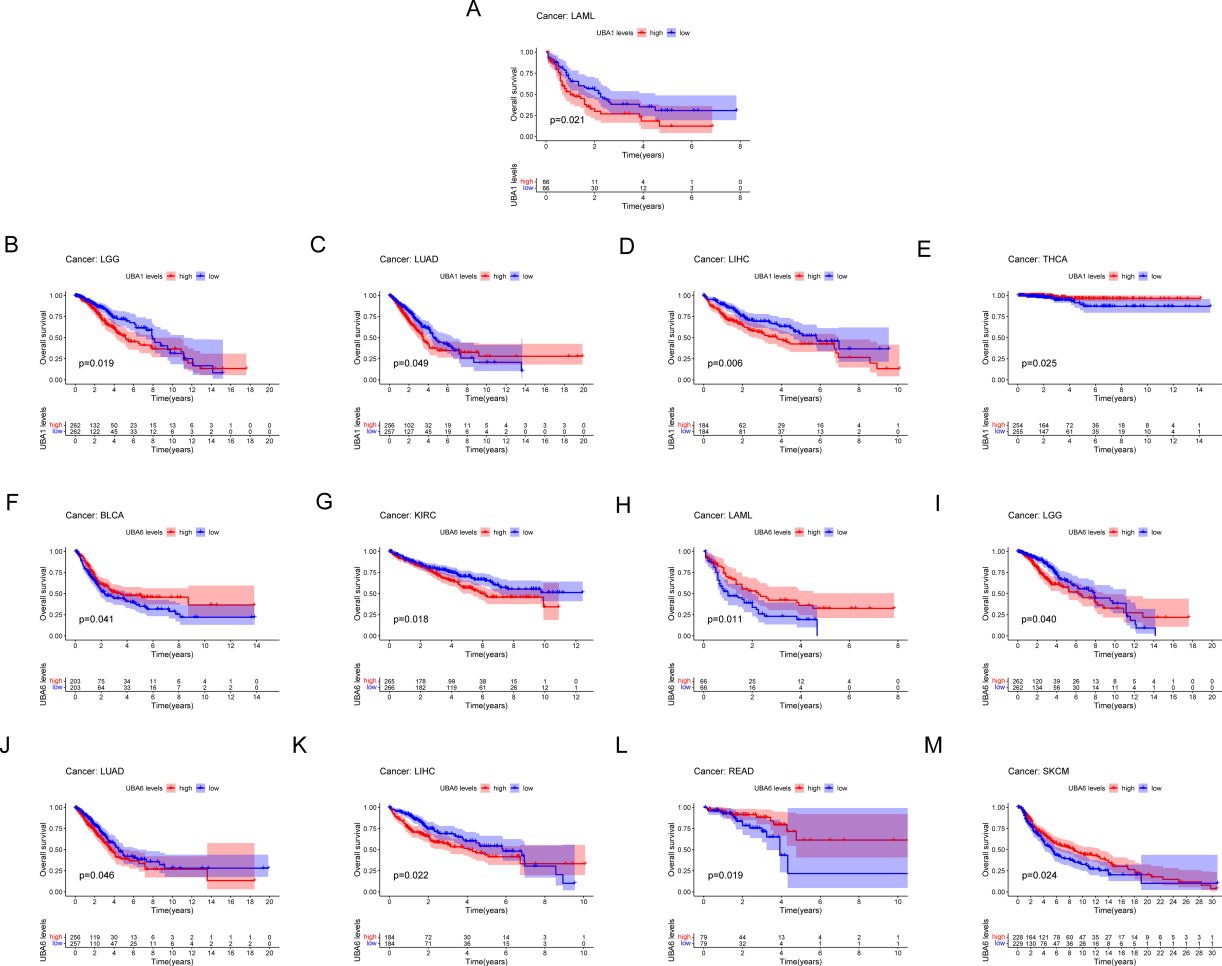
Comparison of Kaplan-Meier survival curves with high and low expression of UBA1/6 in pan-cancer. **(A-E)** OS survival curve of UBA1 in different cancers: LAML, p=0.021; LGG, p=0.019; LUAD, p=0.049; LIHC, p=0.006; THCA, p=0.025。 **(F-M)** OS survival curve of UBA6 in different cancers: BLCA, p=0.041; KIRC, p=0.018; LAML, p=0.011; LGG, p=0.040; LUAD, p=0.046; LIHC, p=0.022; READ, p=0.019; SKCM, p=0.024.

Survival analysis of the TCGA database showed a correlation between UBA1/6 gene expression and the prognosis of several cancers (**Figure 5**), indicating that higher UBA1 expression was associated with bad endpoint in tumor patients with LAML (p=0.021), LGG (p=0.019), LUAD (p=0.049), and LIHC (p=0.006) (**Figures 5A–D**). Higher UBA6 expression was associated with poor OS in tumor patients: KIRC (p=0.018) (**Figure 5G**), LGG (p=0.040) (**Figure 5I**), LUAD (p=0.046) (**Figure 5J**), LIHC (p=0.022) (**Figure 5K**). Interestingly, UBA1 was protective in THCA (p=0.025) (**Figure 5E**), UBA6 in BLCA (p=0.041) (**Figure 5F**), LAML (p=0.011) (**Figure 5H**), READ (p=0.019) (**Figure 5L**) and SKCM (p=0.024) (**Figure 5M**), suggesting that UBA6 may have a protective effect in BLCA, LAML, READ, and SKCM, while UBA1 may also have an oncogenic effect in THCA. Additionally, we also investigated the prognostic value of UBA1/6 in pan-cancer from GEO database, and discovered that elevated UBA1 expression in breast cancer (GSE1456), gastric cancer (GSE14210), and myeloma (GSE24080) is associated with a poorer prognosis, whereas higher UBA1 expression correlates with a more favorable prognosis in colon cancer (GSE1258)., Notably, the relationship between UBA6 expression and prognosis did not reach statistical significance in the aforementioned dataset. (**Supplementary Figure S2**).

**Figure 6:**
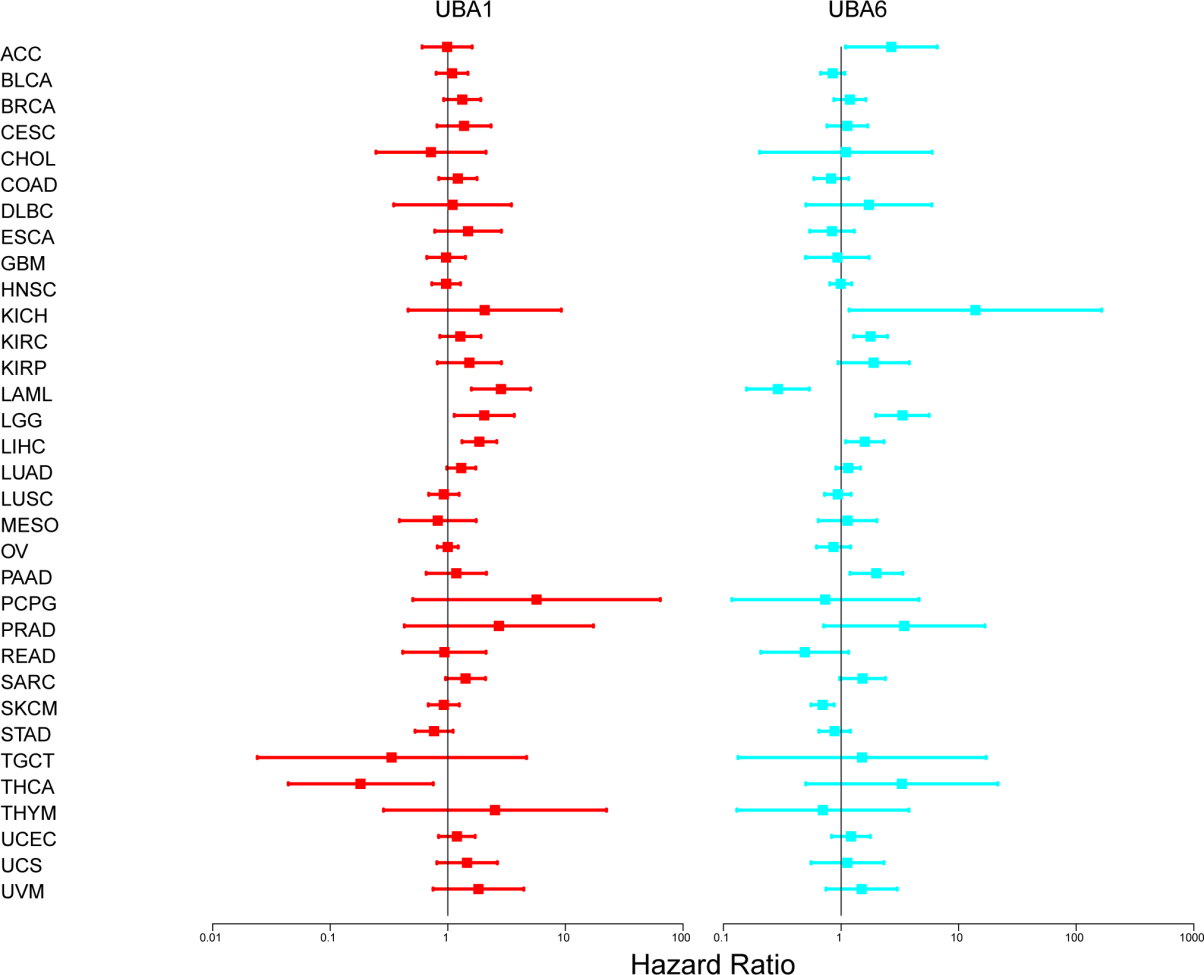
The relationship between UBA1/6 gene expression and total survival of patients with different cancer types. The forest map with the risk ratio and 95% confidence interval of the total survival rate of different cancer types shows the survival advantages and disadvantages of increased UBA1/6 expression. A Univariate Cox proportional risk regression model is used for the correlation test.

We further investigated the prognostic risk of UBA1/6 in pan-cancer by COX analysis (**Figure 6**). Our results showed that UBA1 played an unfavorable role in LAML, LGG and LIHC (HR>1, P<0.05). On the other hand, UBA1 had a protective effect on THCA (HR<1, P<0.05). UBA6 was a detrimental prognostic factor in ACC, KICH, KIRC, LGG, LIHC, and PAAD (HR>1, P<0.05). In contrast, UBA6 was a protective prognostic factor in LAML and SKCM (HR < 1, P < 0.05).

### UBA1/6 mutational profile in pan-cancer based on GSCA analysis

**Figure 7:**
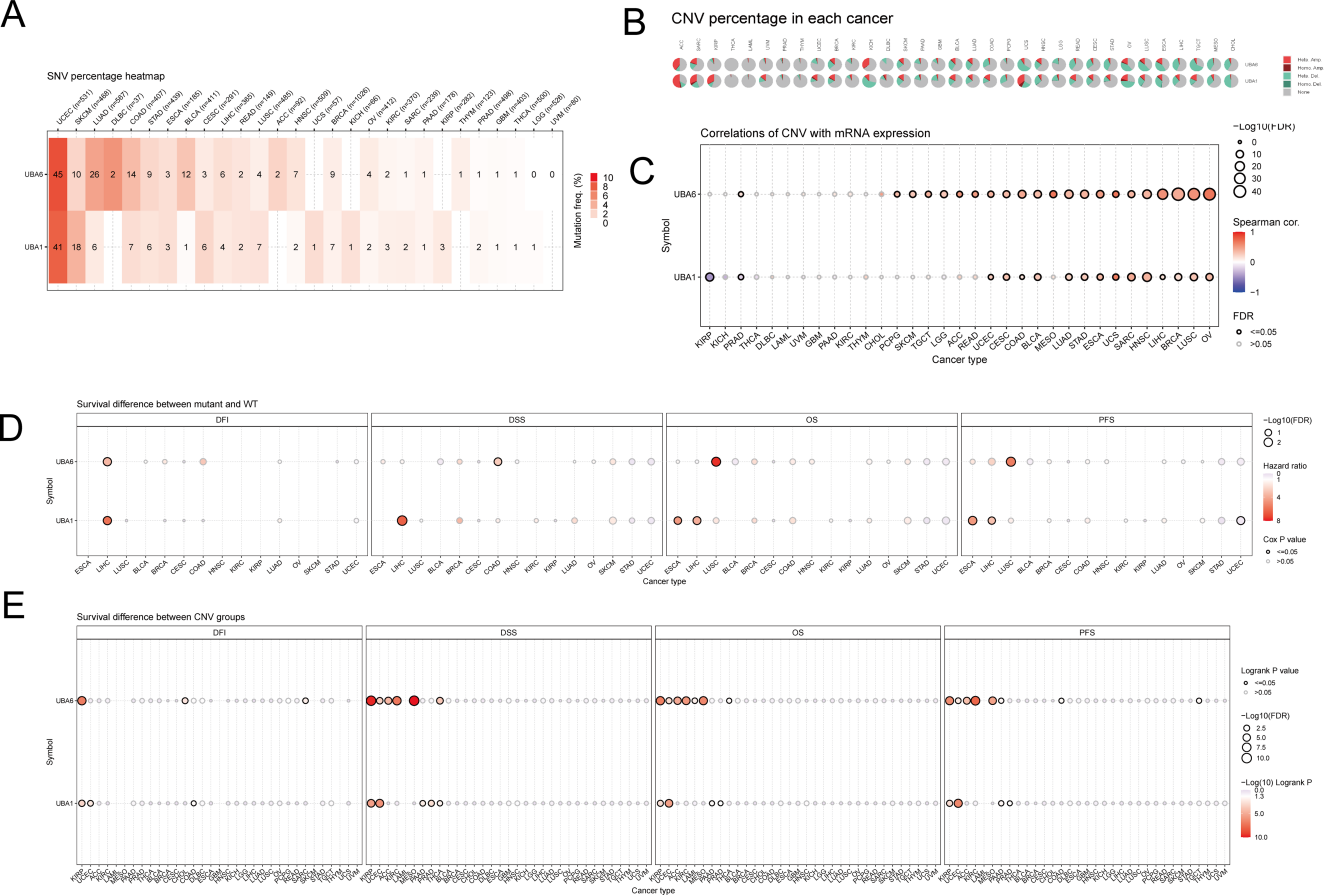
The mutational landscape of UBA1/6 in pan-cancer. **(A)** The SNV percentage heatmap of UBA1/6 in various tumors. **(B)** The CNV percentage pie of UBA1/6 in various tumors. **(C)** The correlations of CNV with UBA1/6 mRNA expression. **(D)** The survival difference between geneset SNV associated UBA1/6 and wild type. **(E)** The survival difference between geneset CNV associated UBA1/6 and wild type. p value less than 0.05 is considered a difference. SNV, single nucleotide variants; CNV, Copy number variations.

Here, we summarized UBA1/6 SNV percentage in 33 cancer types by using the GSCA database, respectively. The results indicated that the highest mutation frequency of UBA1/6 were both seen in UCEC, while the lowest mutation rates were found in UVM (**Figure 7A**). Meanwhile, we also detected the CNV percentage in these tumors, and suggested that the highest heterozygous amplification ratio for UBA1/6 was found in ACC. Besides, a relatively higher heterozygous deletion ratio (>50%) for UBA6 was seen in UCS and TGCT, while UBA1 was relatively higher in KICH (**Figure 7B**). We also explores the association between UBA1/6 CNV and their mRNA expression. Except for KIRP, KICH, THCA, DLBC, LAML, UVM, GBM, PAAD, KIRC, THYM and CHOL, the rest cancer types were statistically significant for the correlation between UBA6 CNV and its mRNA expression. Interestingly, a negative correlation between UBA1 CNV and mRNA was detected in KIRP (**Figure 7C**). Finally, we analyzed the survival difference between UBA1/6 associated gene set SNV groups in the selected cancers, and found that only UBA1 had all statistical significance on OS, PFS, DFS and DFI in LIHC. For UBA1/6 CNV groups, UBA1 had all statistical significance on above four survival indicators only in KIRP and UCEC, however, UBA6 associated survival difference were also seen in ACC, KIRC and MESO (**Figures 7D, E**).

### UBA1/6 expression is associated with tumor mutational load and microsatellite instability

Further analysis revealed that UBA1 expression was positively correlated with TMB in BLCA, BRCA, ESCA, LGG, SARC, STAD and UCEC, but negatively correlated with KIRP and THYM (**Supplementary Figure S3A**). However, UBA6 expression was not associated with TMB in BLCA, BRCA, ESCA, KIRP, and THYM (**Supplementary Figure S3B**). As in **Supplementary Figure S3A**, we also found that UBA1 was positively correlated with MSI in GBM, KIRC, LIHC, LUAD, LUSC, SARC, STAD, TGCT, UCEC, and UVM, but negatively correlated with BRCA, DLBC, HNSC, READ, and THCA. Similarly, a correlation analysis between UBA6 expression and MSI was also performed (**Supplementary Figure S3B**). In ACC, COAD, READ, SARC, STAD and UCEC, UBA6 expression was positively correlated with MSI, while UBA6 expression was negatively correlated with BRCA, DLBC, HNSC and SKCM.

### UBA1/6 expression in pan-cancer in relation to tumor microenvironment

Cancer stem cells play a key role in tumor proliferation, migration, metastasis, epithelial-mesenchymal recurrence, and treatment resistance. Therefore, we explored the association between UBA1/6 expression and stemness scores. For example, UBA1/6 expression was positively correlated with DNAss in DLBC and GBM. Meanwhile, we found that UBA1/6 was negatively correlated with DNAss in TGCT. In addition, UBA1 was significantly positively correlated with DNAss in CHOL, while UBA6 was opposite (**Supplementary Figure S4A**). During the RNAss analysis, we observed that UBA1 was positively correlated with RNAss in ACC, KICH, SARC and STAD. UBA6 was positively correlated with RNAss in various cancers including ACC, LAML, PCPG and STAD, and negatively correlated with RNAss in CHOL and THCA (**Supplementary Figure S4B**). In addition, the results of tumor microenvironment-related scores showed that most UBA1/6 expression was significantly negatively correlated with stromal score, immune score and estimation score, and positively correlated with tumor purity, which was particularly significant in ACC (**Supplementary Figures S4C–F**). Specifically, we could find that UBA1/6 appeared to be consistently negatively correlated with all cancers in these scores. These results suggest that the ability of UBA1/6 to regulate the immune microenvironment varies across cancers.

### UBA1/6 expression is associated with immune subtypes in cancer

We compared the relationship between UBA1/6 expression and immune subtypes through the TISIDB database (**Supplementary Figure S5**). Immune subtypes were classified into six types, including C1 (wound-healing), C2 (IFN-gamma dominant), C3 (inflammatory), C4 (lymphocyte depleted), C5 (immunologically quiet) and C6 (TGF-b dominant). Our analysis showed that UBA6 expression in BRCA, COAD, KIRC and LUAD was significantly correlated with immune subtypes. UBA1 expression in BRCA, KIRC and LUAD was strongly correlated with immune subtypes, while expression in COAD immune subtypes was not statistically significant. Interestingly, in LUAD, for example, UBA1 showed high expression in C1 and C3 immune subtypes, while UBA6 had the highest expression on C2 type. Based on these results, we concluded that the diversity of UBA1/6 expression in the immune subtypes of specific tumor cancers may influence the endpoint of these tumors in varying degrees.

### Correlation of UBA1/6 expression with tumor immune infiltration cells and immune checkpoints

**Figure 8:**
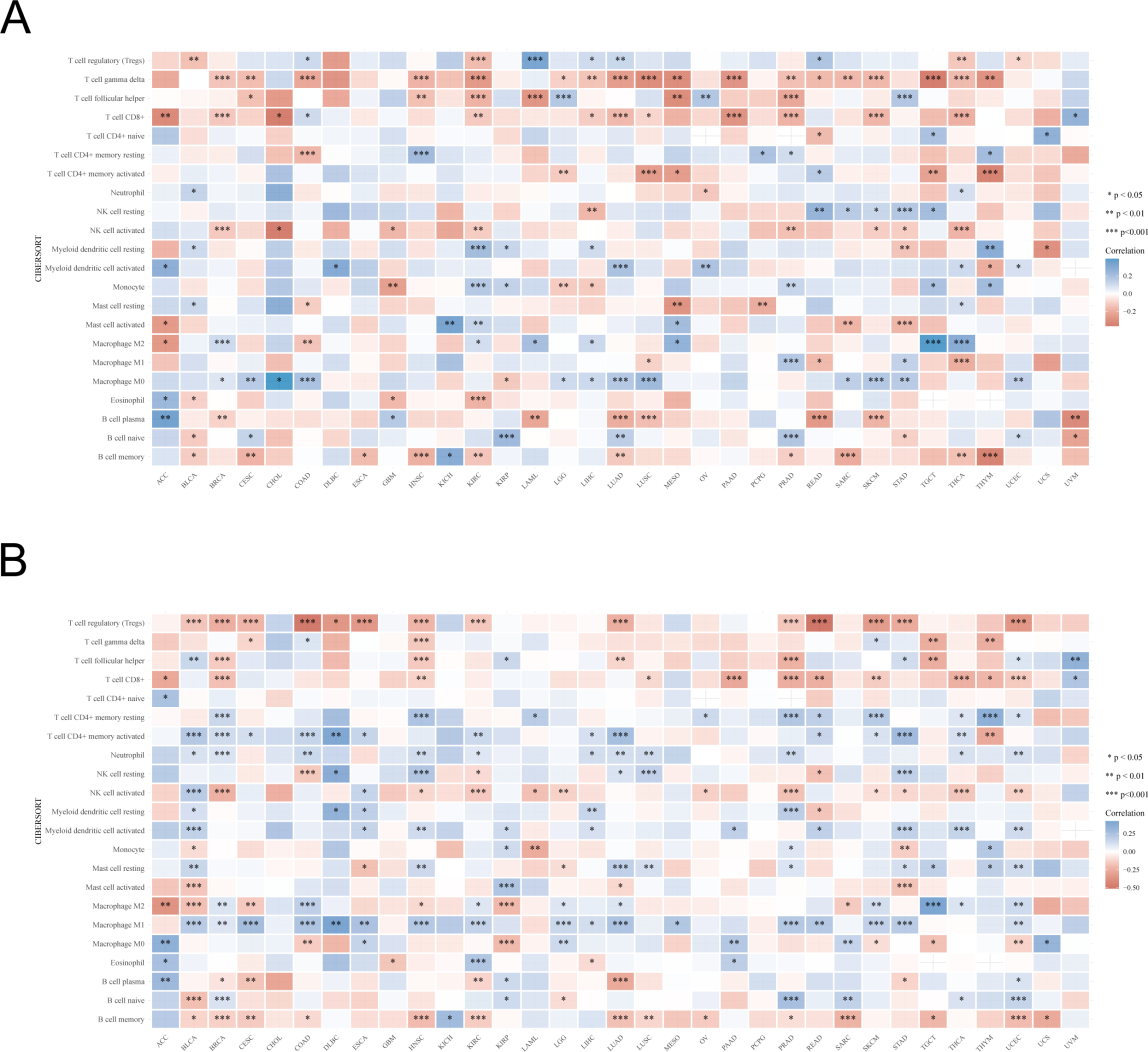
The relationship between UBA1/UBA6 expression and immune infiltration cells. **(A, B)** The correlation between UBA1 expression and immune infiltration cells of 33 cancer types. Red represents a positive correlation, and blue represents a negative correlation. *p <0.05, **p <0.01, ***p <0.001.

We investigated the relationship between UBA1/6 expression and immune cell infiltration using CIBERSORT algorithm (**Figure 8**). For different cancers, there were similarities and differences in the correlation between UBA1/6 expression and immune cells infiltrating the tumor. We found that in most tumors, high UBA1 expression was positively correlated with T cell gamma delta, T cell CD8+, and B cell memory cell infiltration and negatively correlated with Macrophage M0 cell infiltration. However, in most tumors, high UBA6 expression was positively correlated with T cell regulatory (Tregs), T cell CD8+, NK cell activated, B cell memory cell infiltration, and negatively related with T cell CD4+ memory resting, T cell CD4+ memory activated, Neutrophil, Myeloid dendritic cell activated, and Macrophage M1 cell infiltration. In particular, we also found that UBA6 expression in CHOL was not significantly correlated with 22 immune infiltrating cells. And the expression of UBA1 in DLBC and ESCA was not significantly correlated with 21 immune infiltrating cells. Interestingly, in COAD, high UBA6 expression was positively correlated with T cell regulatory (Tregs), Macrophage M0 immune cell infiltration and negatively correlated with T cell gamma delta, Macrophage M2, however, high UBA1 expression correlated opposite with these four immune cell infiltrates. Additionally, we also examined the abundance of these two members in immune cells using a single-cell database to further assess their association with immune cells (**Supplementary Figure S6**).

**Figure 9:**
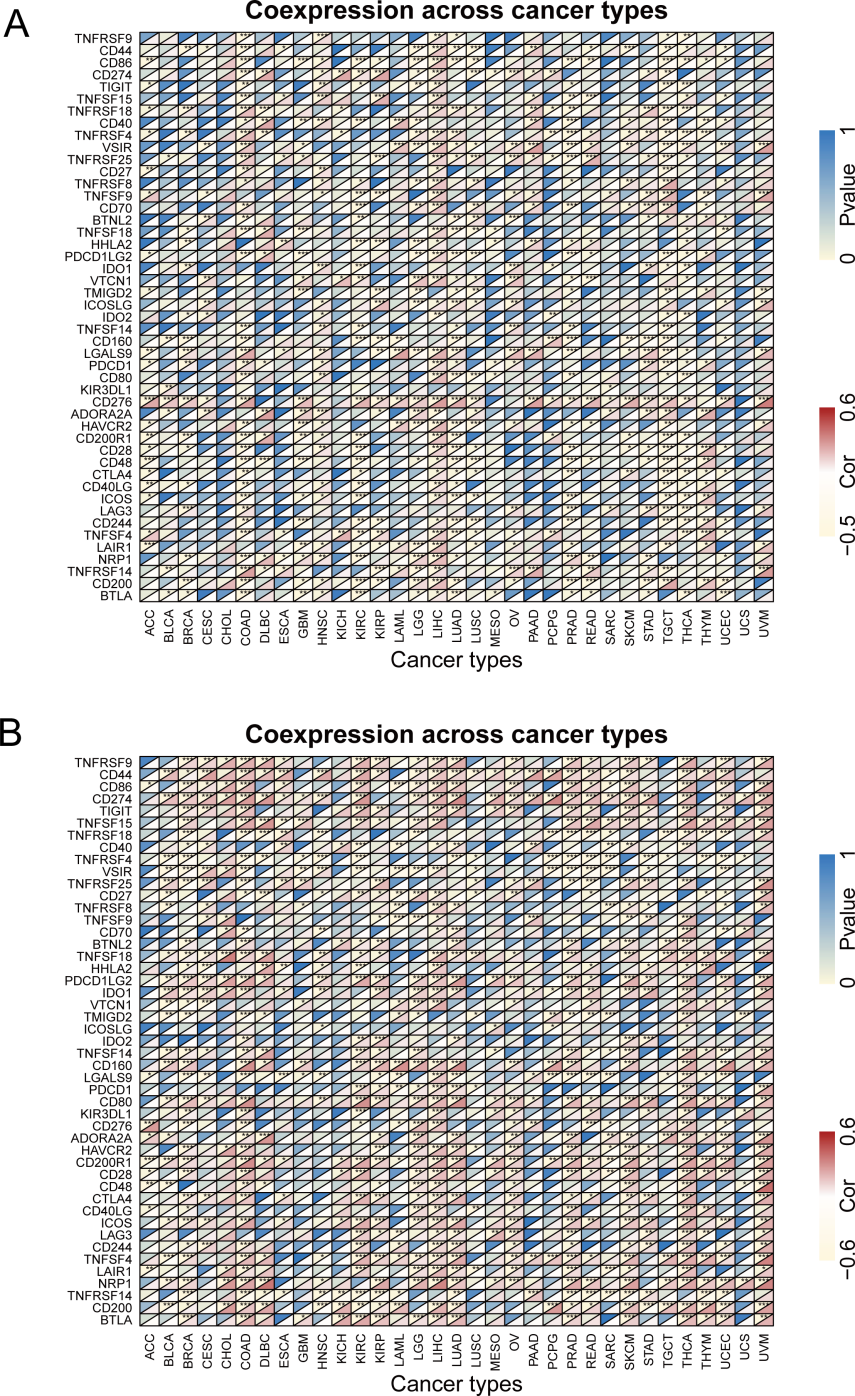
The relationship between UBA1/UBA6 and immune checkpoints. **(A)** Heatmap showing the relationship between UBA1 and known immune checkpoints. **(B)** Heatmap showing the relationship between UBA6 and known immune checkpoints. The top left triangle represents the P-value, and the bottom right triangle represents the correlation coefficient. *p <0.05, **p <0.01, ***p <0.001, ****p <0.0001.

In addition, co-expression analysis using TCGA database could reveal the correlation between UBA1/6 expression and immune checkpoints in pan-cancer (**Figure 9**). As shown in **Figure 9A**, UBA1 was negatively correlated with these representative immune markers in COAD, GBM, KIRC, LUAD, PRAD, TGCT and THCA, while in LIHC, its expression showed a positive correlation with most of checkpoints. In contrast, UBA6 expression was positively correlated with these immune checkpoints in most cancers, including COAD, DLBC, KIRC, KIRP, LIHC, LUAD, THCA and UVM (**Figure 9B**). Besides, we also found that only CD276 was positively associated with UBA1 in most cancers, however, there are many other immune checkpoints like CD44, CD86 and CD274 illustrating a positive correlation with UBA6. CD276 expression was negatively with UBA6 only in a few cancers such as BRCA, COAD and READ. The opposite relationship between UBA1 and UBA6 in immune infiltration may account for the different prognosis of cancer patients.

### Correlation analysis of UBA1/6 expression and drug sensitivity

**Figure 10:**
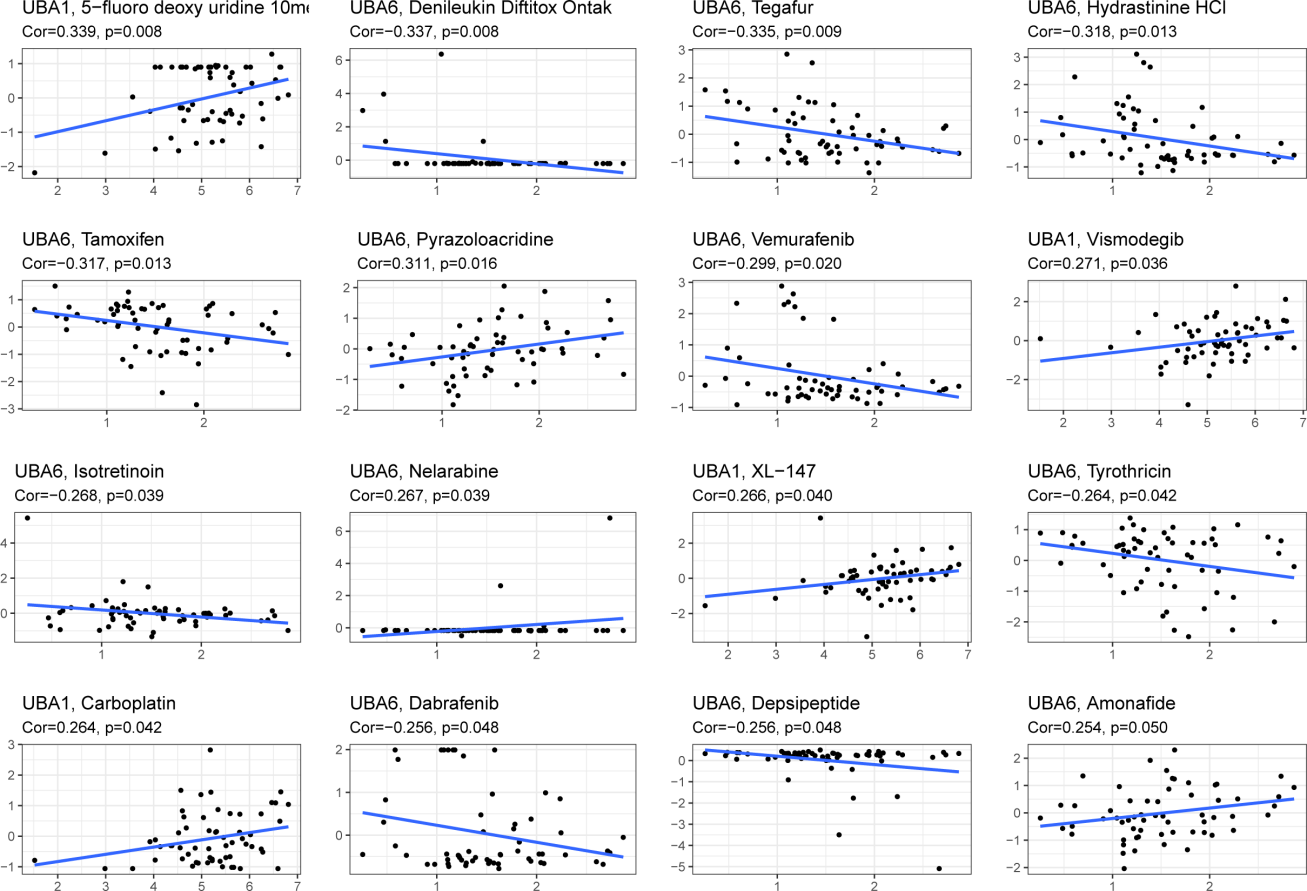
The expression of UBA1/6 in CellMiner was significantly correlated with the first 16 items of anticancer drug sensitivity.

From **Figure 10**, we can see that the higher the expression of UBA1, the higher the drug sensitivity of 5-fluoro deoxy uridine, Vismodegib, XL-147 and Carboplatin (P<0.05). Additionally, we also detected the association between UBA6 expression and representative drugs, and found that UBA6 expression was negatively correlated with the drug sensitivity of Tamoxifen, Vemurafenib, Isotretinoin, Tyrothricin, Dabrafenib, Depsipeptide and so on (P<0.05), meanwhile, the expression of UBA6 was significantly positively correlated with the drug sensitivity of Pyrazoloacridine, Nelarabine and Amonafide (P<0.05). These above findings might provide new and potential direction on clinical chemotherapy through targeting UBA1/6.

### Protein-protein interaction network and functional enrichment analysis

**Figure 11:**
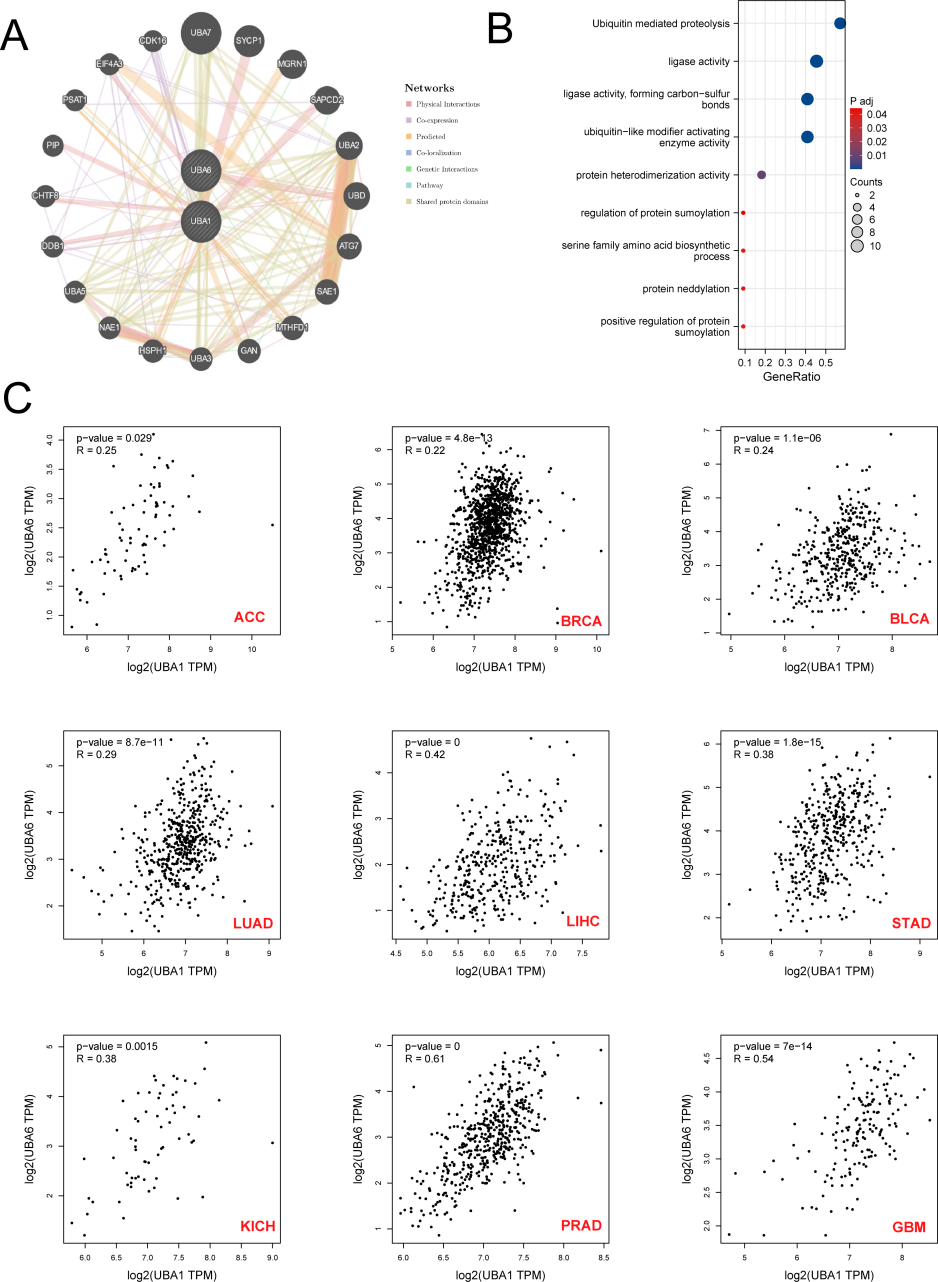
The Prediction of the function and pathway of UBA1/UBA6 related molecules. **(A)** Protein-protein interaction (PPI) network of UBA1/UBA6. **(B)** Kyoto Encyclopedia of Genes and Genomes enrichment analysis of UBA1/UBA6 related molecules. **(C)** The correlation relationship between UBA1 and UBA6 in various types of tumors. p value less than 0.05 is considered a difference.

To explore the proteins interacting with UBA1/6, we used GeneMANIA to construct and visualize the PPI network. Among the 20 proteins interacting with UBA1/6, UBA7, SYCP1, MGRN1, SAPCD2 and UBA2 were the most closely related proteins (**Figure 11A**). The functions of UBA1/6 and these proteins were mainly related to Ubiquitin mediated proteolysis, ligase activity ligase activity, forming carbon-sulfur bonds and ubiquitin-like modifier activating enzyme activity processes (**Figure 11B**). Finally, we analyzed the association between UBA1 and UBA6, and found that they were positively related with most cancers, especially PRAD and GBM, which indicated that they may modulate each other in the ubiquitination system to make it more sophisticated (**Figure 11C**).

## Discussion

The ubiquitin-activating enzyme E1 represents the initial enzyme in the ubiquitination cascade. Current research predominantly concentrates on the two E1 isoforms, UBA1 and UBA6, which play crucial roles in modulating major downstream ubiquitination processes within the human body (35). In our study, multi-omics data revealed the expression patterns, clinical stage, prognosis, mutation landscape and immune infiltration of UBA family using TCGA and GEO database, and the results indicated UBA family members have inconsistent characteristics in different tumors, which may be an important factor leading to tumor heterogeneity. Previous researches have shown that tumor heterogeneity facilitates the adaptation of tumor cells to alterations in the tumor microenvironment, thereby enhancing tumor resistance and progression (36, 37). Therefore, targeting UBA family members could represent a potentially pivotal strategy for addressing tumor heterogeneity and overcoming drug resistance in refractory tumors.

Researches have shown that the expression of UBA1 and UBA6 was closely related to tumor occurrence and progression (38). Many tumors exhibit abnormal protein ubiquitination patterns and cell cycle disorders (39). For example, studies on fruit flies have shown that partial deletion of UBA1 affects cell apoptosis, while complete deletion leads to cell cycle arrest and excessive tissue growth in a non-cellular manner (40). These studies suggest that UBA1/6 may be involved in tumor progression by regulating cell cycle. Therefore, inhibiting E1 activity can inhibit some downstream ubiquitination related to tumors. In our study, we found that UBA1/6 showed a high expression trend in various malignant tumors, especially BRCA, CHOL, COAD, etc. Moreover, the expression of UBA1/6 was also related to tumor stages. However, further research is needed on how UBA1/6 affects tumor progression and stage.

The tumor stroma comprises various tumor-associated fibroblasts, macrophages, and infiltrating lymphocytes, all of which play a critical role in the malignant transformation of tumors. Numerous previous studies have reported that ubiquitination enzymes are involved in the regulation of tumor immune microenvironment to alter immune efficacy (41–43). Hanawalt found that the reduction of UBA1 phosphorylation in macrophages can alleviate nucleotide excision repair defects in macrophages (44). Interestingly, in our study, we found that UBA1/6 was associated with multiple immune cell infiltration, including T cells, NK cells, and macrophages. Cancer progression is usually associated with immunosuppressive tumor microenvironment (TME) (45). For example, when CD8^+^T cells are reduced or dysfunctional, tumor cells cannot be killed (46). In addition, Treg cells in tumor tissue increase and become functionally sexual maturity, leading to the formation of immunosuppressive TME (47). Herein, we also found that the expression of UBA1 was positively correlated with CD8-T cells in ACC, BRCA, PAAD, PRAD, etc., and UBA6 expression is closely related to Treg cells across various tumors. Therefore, controlling the expression of UBA1/UBA6 in tumors might regulate tumor TME and promote the killing effect of immune cells on tumors.

In summary, our study identified the potential driving role of UBA family in pan-cancer, especially BRCA, COAD, LUAD and KIRC. Firstly, we conducted a comprehensive analysis of the clinical heterogeneity of the UBA family in pan-carcinoma using the TCGA database, examining factors such as gene expression, cancer stage, drug sensitivity, mutation landscape, and prognosis. Subsequently, we found that UBA1/6expression was closely related to immune infiltration components, which might be possible to improve the effect of immunotherapy on tumors. However, to some extent, our research still has certain limitations. For example, we only have data analysis results and lack clinical experimental validation. In the future, it is imperative to conduct an in-depth exploration and analysis of the regulatory mechanisms of the UBA family within the tumor immune microenvironment in order to elucidate the prevailing conditions of clinical immune insensitivity. More importantly, we urgently need high-throughput sequencing and biological experiments focused on UBA1- and UBA6-specific tumors to address tumor heterogeneity effectively.

## Data Availability

The original contributions presented in the study are included in the article/**Supplementary Material**. Further inquiries can be directed to the corresponding authors.
