# Supplementary material for: Multi-omics analysis identifies UBA family as potential pan-cancer biomarkers for tumor prognosis and immune microenvironment infiltration
Source: Front Immunol. 2025 Feb 17;16:1510503. doi: 10.3389/fimmu.2025.1510503 (PMC11880792; doi:10.3389/fimmu.2025.1510503)
Supplement: Supplementary file 1 [file DataSheet1.docx]

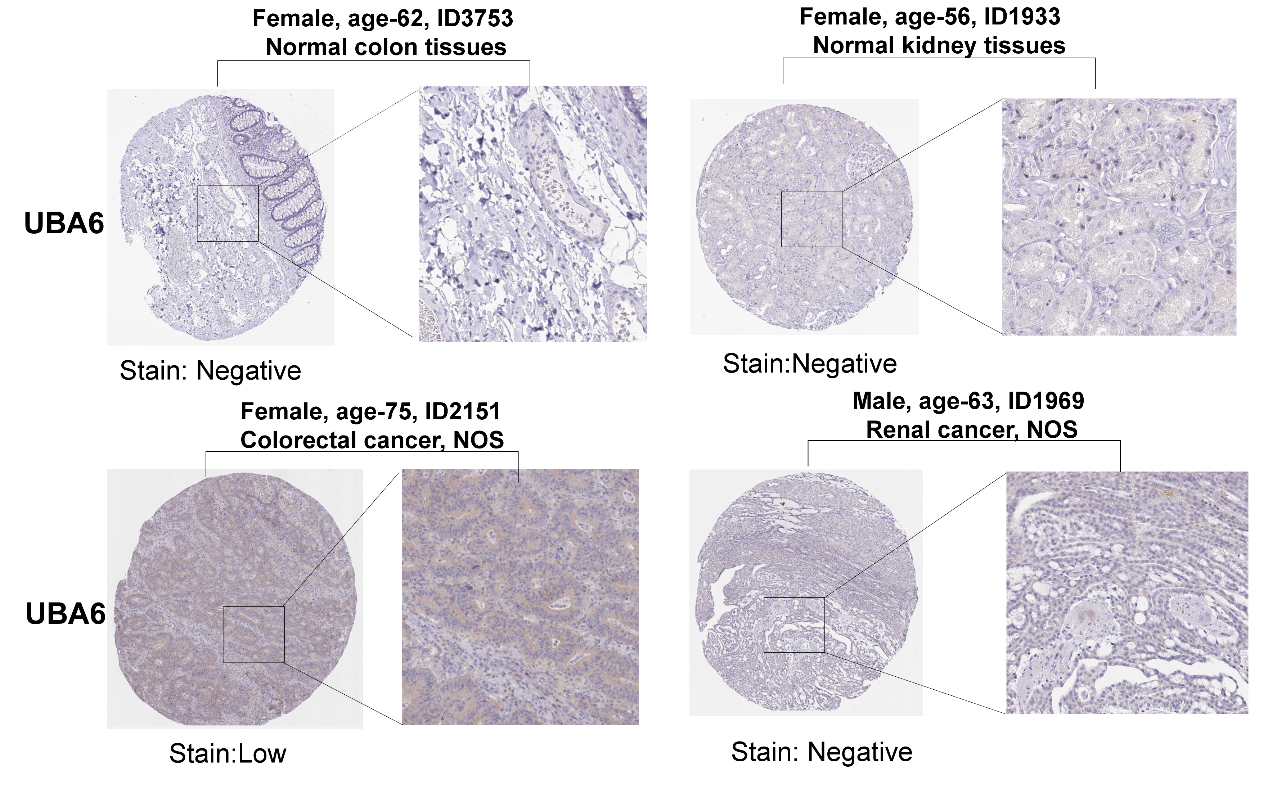


**Figure S1**

**
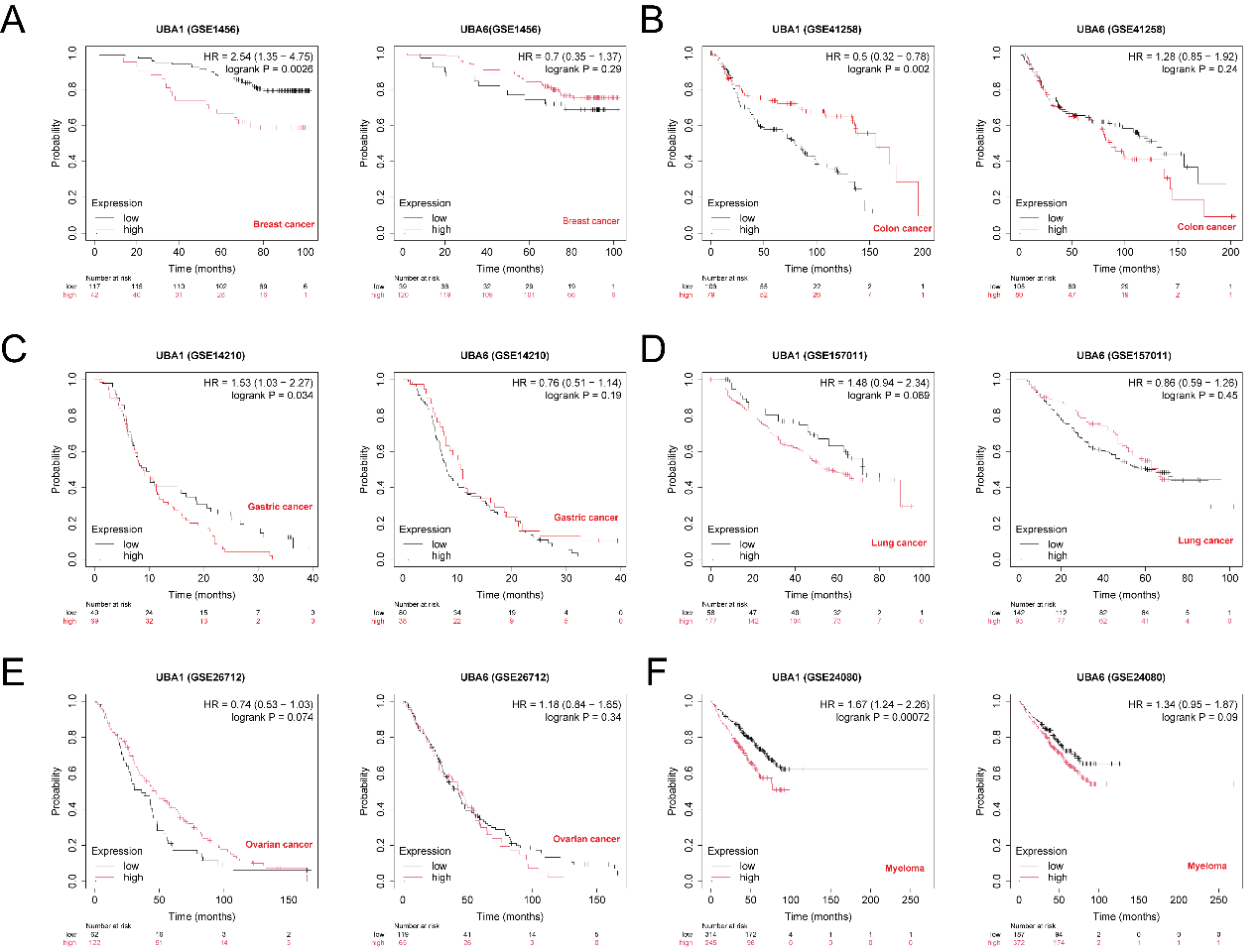
**

**Figure S2**

**
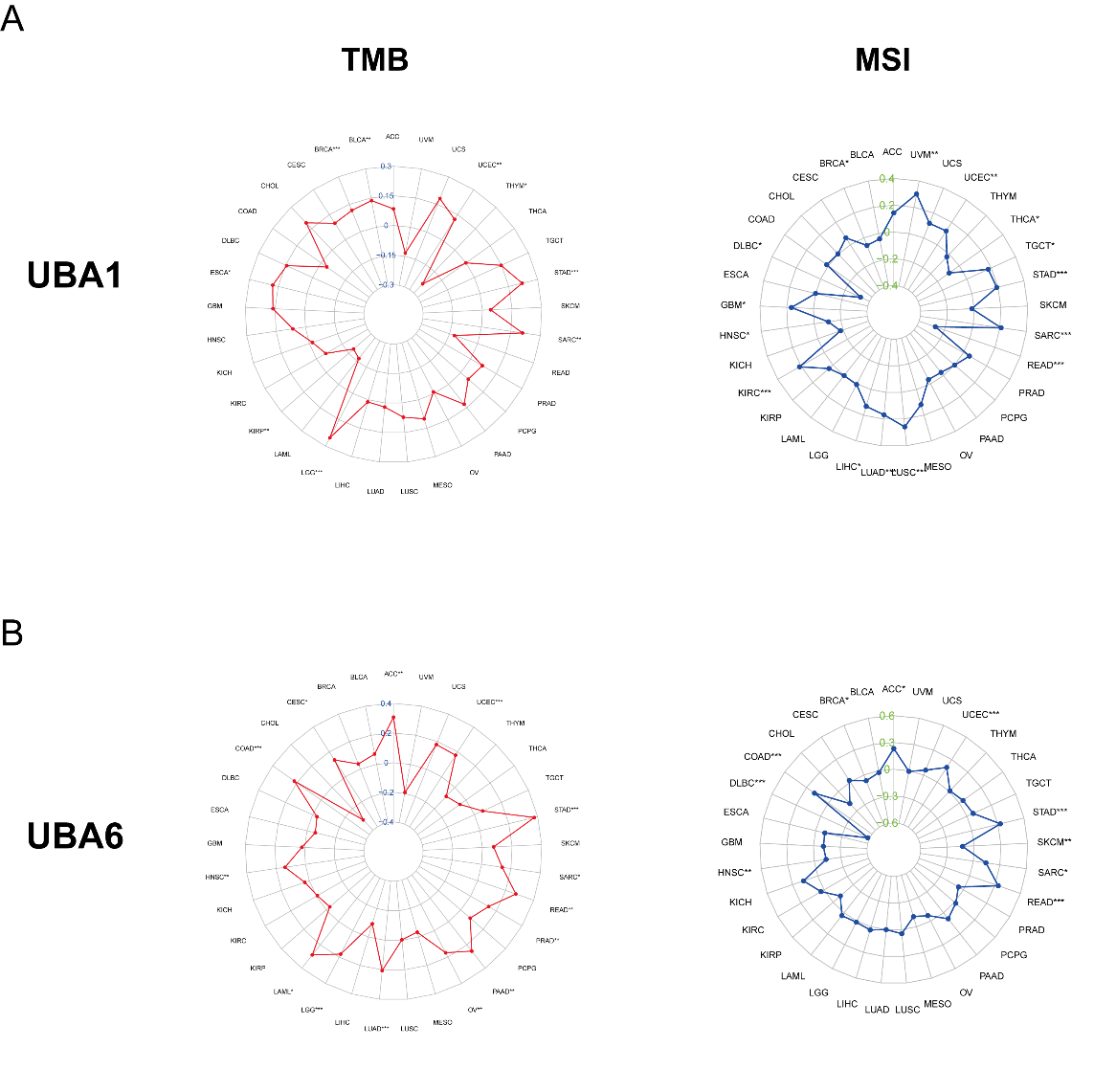
**

**Figure S3**

**
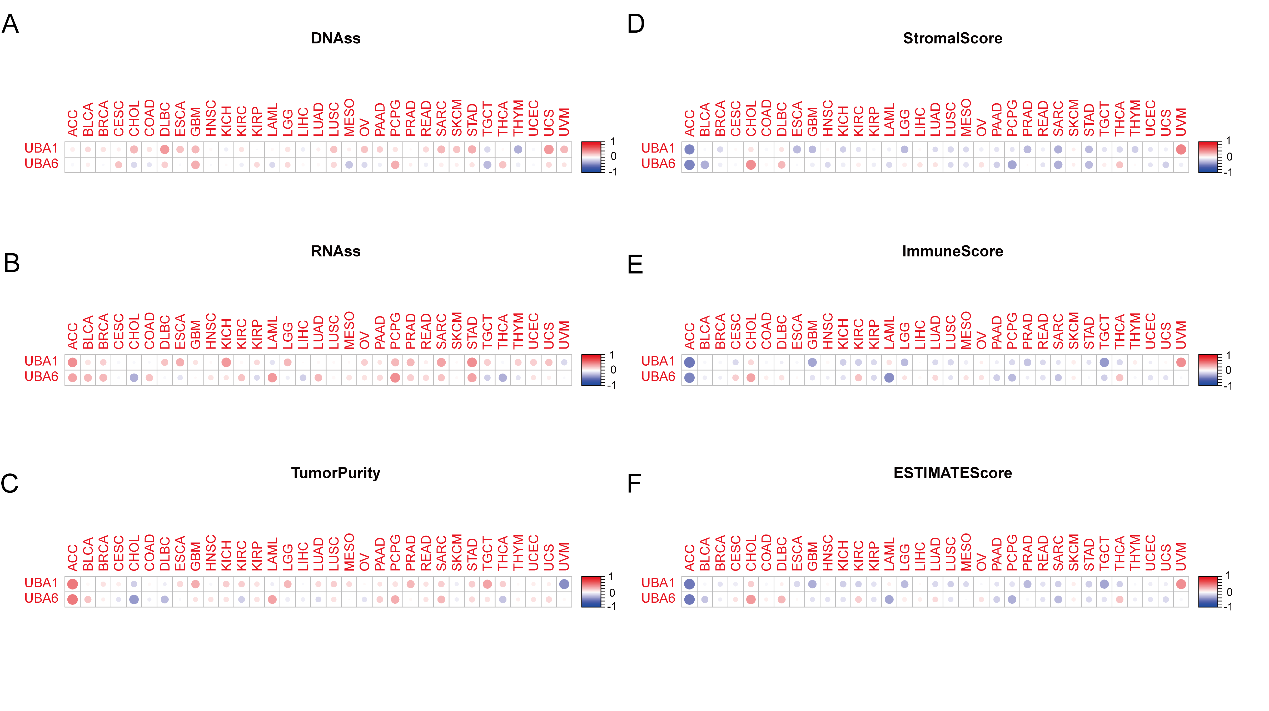
**

**Figure S4**

**
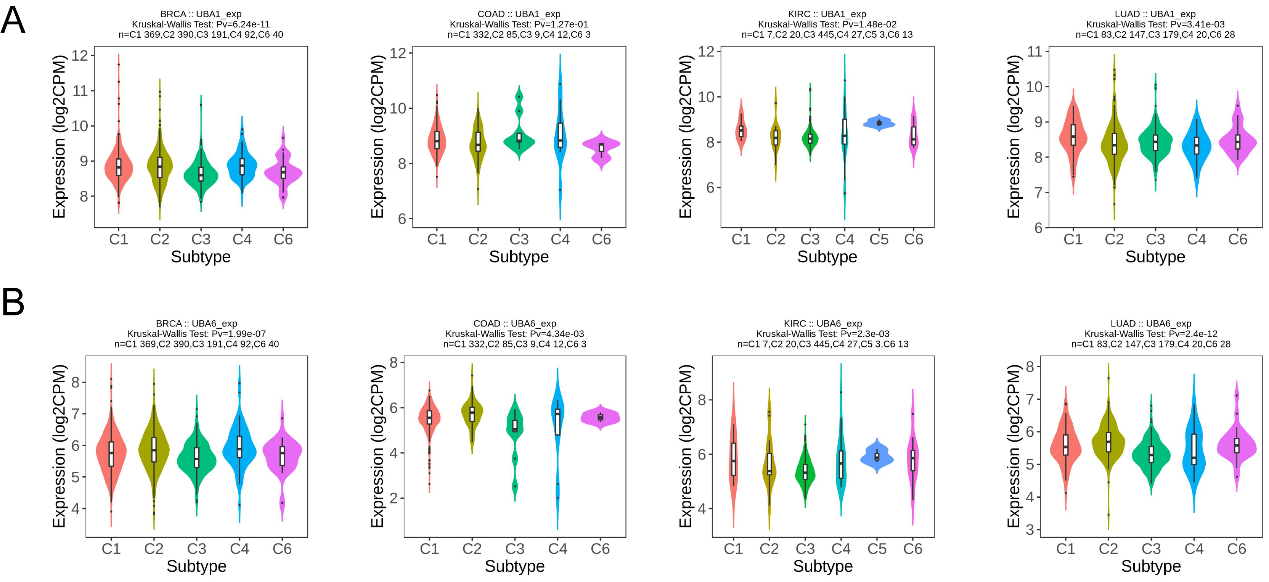
**

**Figure S5**


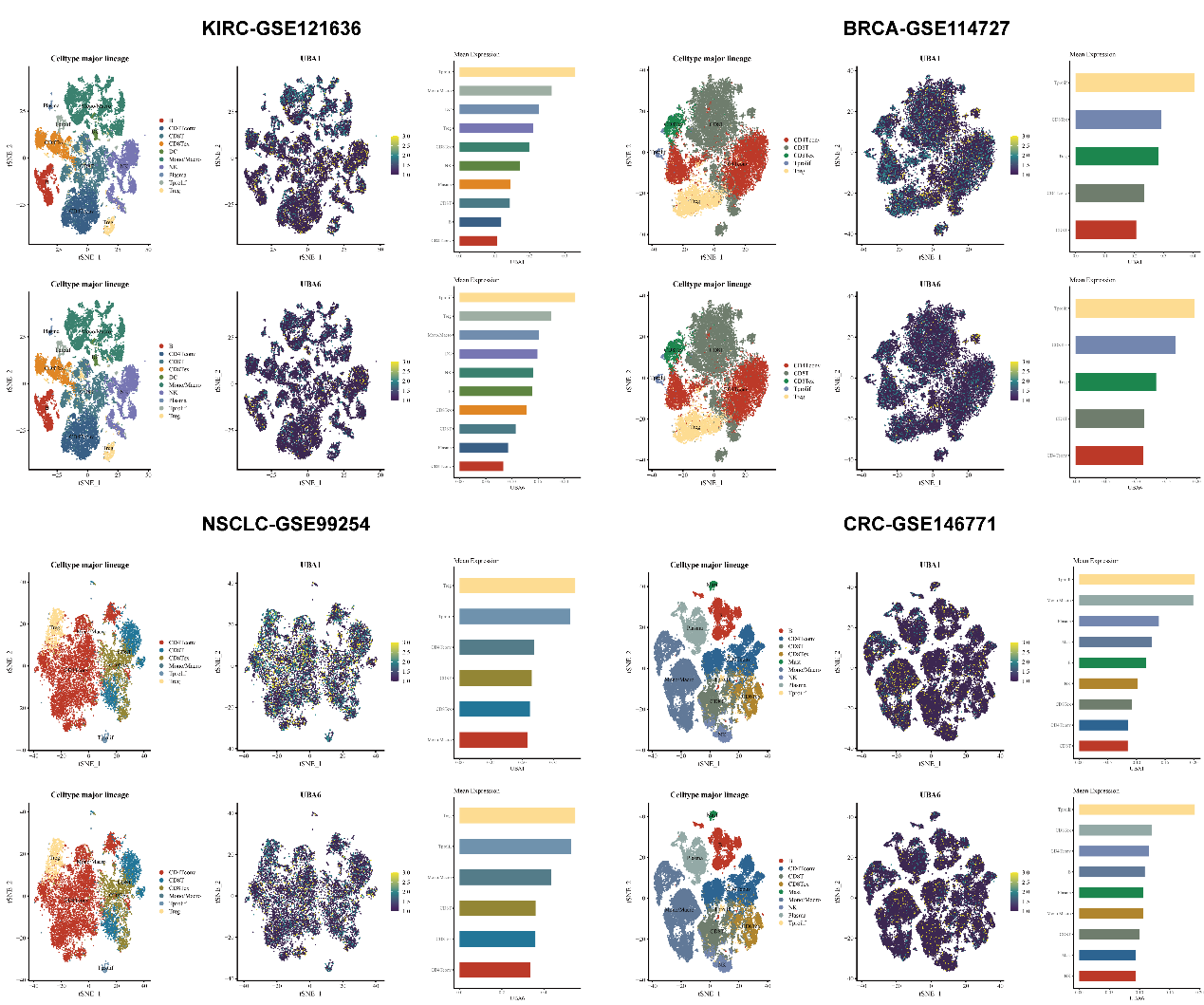
**Figure S6**

**Figure S1:**

The protein level of UBA6 expression in colorectal cancer and renal cancer tissues and matched normal tissues from the HPA database.

**Figure S2：**

The prognosis landscape of UBA1/6 from various GEO database. p value less than 0.05 is considered a difference.

**Figure S3:**

Correlation between UBA1/6 based on TCGA database and TMB and MSI. (A) In UBA1. (B) In UBA6. * p <0.05, * * p <0.01 and * * * p <0.001.
**Figure S4:**

The relationship between UBA1/6 expression and tumor microenvironment factors and pan-cancer stem cell score. The DNAs (A), RNAs (B), tumor purity (C), matrix score (D), immune score (E), and estimated score (F) associated with UBA1/6 were shown.

**Figure S5:**

UBA1/6 genes expression level of different immune subtypes in specific four cancer types. (A) In BRCA. (B) In COAD. (C) In KIRC. (D) In LUAD. The x-axis represents the immune subtype, and the Y-axis represents gene expression. * p <0.05; ** p <0.01; *** p <0.001. C1: wound healing, C2: IFN-gamma dominant, C3: inflammatory, C4: lymphocyte depleted, C5: immunological quiet, and C6: TGF-beta dominant.

**Figure S6:**

The Correlation between UBA1/6 and immune infiltration in BRCA, CRC, KIRC and NSCLC single-cell database. The t-SNE plot and bar chart of single-cell clustering illustrating the expression distribution of UBA1/6 in different cells from GSE121636, GSE114727, GSE99254 and GSE146771 database.
